# STRESS AND DEATH: GLUCOCORTICOIDS AND SURVIVAL IN A WILD PRIMATE MODEL OF AGING

**DOI:** 10.1093/geroni/igad104.0198

**Published:** 2023-12-21

**Authors:** Fernando Campos

**Affiliations:** University of Texas, San Antonio, San Antonio, Texas, United States

## Abstract

Chronic exposure to stressors has been linked with a wide range of detrimental effects on health in humans, laboratory animals, and a few wild animal populations. Glucocorticoids (GCs) mediate stress responses, and consequently GC levels are regularly measured in humans and other animals as biological markers of stress. However, no tests in humans or in natural populations of animals have yet established clear connections between environmental stressors, chronically elevated GCs, and shortened lifespan. To fill this gap, we used longitudinal data on wild female baboons to investigate the relationship between GC levels and survival—the single greatest determinant of variation in evolutionary fitness among female baboons. Using 14,173 GC measurements from 242 wild adult female baboons over 1634 female years, we document a powerful link between GCs and survival: females with relatively high current GCs or high lifelong cumulative GCs face an elevated risk of death. A hypothetical female who maintained GCs in the top 90% for her age across adulthood would be expected to lose 5.4 years of life relative to a female who maintained GCs in the bottom 10% for her age. Hence, differences among individuals in HPA axis activity provide valuable prognostic information about disparities in lifespan. Together, our results both support the value of GCs as a window into health, fitness, and aging and suggest that they may mechanistically contribute to the established link between sociality and lifespan.

